# Effects of Phytochemically Characterized Extracts From *Syringa vulgaris* and Isolated Secoiridoids on Mediators of Inflammation in a Human Neutrophil Model

**DOI:** 10.3389/fphar.2018.00349

**Published:** 2018-04-11

**Authors:** Marta Woźniak, Barbara Michalak, Joanna Wyszomierska, Marta K. Dudek, Anna K. Kiss

**Affiliations:** ^1^Department of Pharmacognosy and Molecular Basis of Phytotherapy, Medical University of Warsaw, Warsaw, Poland; ^2^Centre of Molecular and Macromolecular Studies of Polish Academy of Sciences, Lodz, Poland

**Keywords:** *Syringa vulgaris*, Oleaceae, secoiridoids, neooleuropein, pro-inflammatory mediators, neutrophils

## Abstract

**Aim of the study:** The aim of the present study was to investigate the effects of phytochemically characterized extracts connected with the traditional use (infusions and ethanolic extracts) of different parts of *Syringa vulgaris* (common lilac) on the pro-inflammatory functions of neutrophils. Active compounds were isolated from the most promising extract(s) using bioassay-guided fractionation, and their activity and molecular mechanisms of action were determined.

**Methods:** The extracts were characterized using a HPLC-DAD- MS^n^ method. The effects on ROS, MMP-9, TNF-α, IL-8, and MCP-1 production by neutrophils were measured using luminol-dependent chemiluminescence and enzyme-linked immunosorbent assay (ELISA) methods. The effects on p38MAPK, ERK1/2, JNK phosphorylation, and NF-*k*B p65 translocation were determined using western blots.

**Results:** The major compounds detected in the extracts and infusions belong to structural groups, including caffeic acid derivatives, flavonoids, and iridoids. All extracts and infusions were able to significantly reduce ROS and IL-8 production. Bioassay-guided fractionation led to the isolation of the following secoiridoids: 2″-epiframeroside, oleonuezhenide, oleuropein, ligstroside, neooleuropein, hydroxyframoside, and framoside. Neooleuropein appeared to be the most active compound in the inhibition of cytokine production by attenuating the MAP kinase pathways.

**Conclusion:** The present study demonstrated that common lilac, which is a traditionally used medicinal plant in Europe, is a valuable source of active compounds, especially neooleuropein.

## Introduction

The Oleaceae family encompasses 25 genera and ~600 species. Members of this family include trees, shrubs, or woody climbers with almost worldwide distribution from northern temperate to southern subtropical regions, with the exception of the Antarctic. Genera include *Olea* (20 spp.), *Forsythia* (7–11 spp.), *Fraxinus* (40–70 spp.), *Syringa* (20–30 spp.), *Osmanthus* (15–30 spp.), *Jasminum* (200–300 spp.), *Chionanthus* (80–100 spp.), and *Ligustrum* (40–50 spp.) (Jensen et al., 2002; Evans, 2009). In Central-Eastern Europe, only a few representatives of the Oleaceae family are found: *Fraxinus exelsior* L., *Ligustrum vulgare* L., *Syringa vulgaris* L., and *Forsythia* sp. Moreover, *Olea* (leaves) and *Fraxinus* (leaves) have monographs in the European Pharmacopoeia as well as European Agency EMEA monographs. The *Syringa* genus is distributed in Eurasia, which is subdivided into western (Central and Southeast Europe) and eastern zones (Central to East Asia) (Wallander and Albert, 2000). Most of the species are found in Asia, while only *S. vulgaris* and *S. josikaea* are found in Europe (Jensen et al., 2002; Lendvay et al., 2016). *S. vulgaris* L., known as the common lilac, is native to the Balkan peninsula and is widely cultivated as an ornamental plant in Europe. In Poland, it has been naturalized since the sixteenth century, where it is cultivated but also found in the wild (Witkowska-Zuk, 2013). In European ethnopharmacology, the common lilac inflorescence or leaves in the form of an infusion or alcoholic extract were used to treat gout, rheumatism and muscle/joint aches (Fournier, 1948; Hanlidou et al., 2010; Jarić et al., 2015; Sõukand and Pieroni, 2016). In Poland, Bulgaria, Italy, and Greece, the flowers, leaves, bark, or fruits in the forms of infusions, decoctions, or alcoholic extracts were used as antipyretics and to treat cold, cough, etc. (Fournier, 1948; Kuznicka, 1986; Kuzniewski and Augustyn-Puziewicz, 1986; Anioł-Kwiatkowska et al., 1993; Leporatti and Ivancheva, 2003; Hanlidou et al., 2010). Locally, different parts of the plant are used to treat toothaches, gastrointestinal disorders, skin wounds, and other ailments (Kuznicka, 1986; Kuzniewski and Augustyn-Puziewicz, 1986; Anioł-Kwiatkowska et al., 1993; Agelet and Vallès, 2003; Hanlidou et al., 2010; Papp et al., 2014). Interestingly, in North American materia medica (pharmacognosy) textbooks, the antipyretic use of the leaves, flowers, and fruits of the common lilac was mentioned (Lyle, 1897; Remington and Woods, 1918), and native Americans used to chew the bark or leaves for treating a sore mouth (Moerman, 1998). The Asian species are also used in traditional medicine to treat bronchial disease [*Syringa reticulata* (Blume) H. Hara leaves], lung and heart diseases (*Syringa pinnatifolia* Hemsl. stem), tooth pain (*Syringa vetulina* Kom. Bark), and as antipyretics (*Syringa afghanica* C.K. Schneid. leaves) (Park et al., 1999; Takenaka et al., 2002; Machida et al., 2009; Su et al., 2016).

Scientific data supporting the traditional use of *S. vulgaris* are connected with its antioxidant, anti-inflammatory, anti-nociceptive and antipyretic properties (Bálinet, 1971; Tóth et al., 2015, 2016; Dudek et al., 2017). Moreover, information about the single compounds responsible for each of these activities is scarce.

The aim of the present study was to investigate the effects of phytochemically characterized (HPLC-DAD-MS^n^) extracts connected with the traditional use (infusions and ethanolic extracts) of different parts of the common lilac (bark, fruits, leaves and flowers) on the pro-inflammatory functions of neutrophils, such as reactive oxygen species (ROS) production, metalloproteinase-9 (MMP-9), interleukin 8 (IL-8), and tumor necrosis factor (TNF-α) release. We isolated active compounds from the most promising extracts using bioassay-guided fractionation. Finally, we focused on the activity and molecular mechanisms of action of the isolated compounds that were able to regulate neutrophil inflammation. The biological activities of single compounds were compared with oleuropein and ligstroside, characteristic compounds from the Oleacea family (Jensen et al., 2002), and with positive control clarithromycin, a macrolide antibiotic, which has been shown to inhibit the pro-inflammatory function of neutrophils (Simpson et al., 2008; Cervin et al., 2009).

## Materials and methods

### Chemicals and general experimental procedures

Hanks' balanced salt solution (HBSS), RPMI 1640, f-MLP (formyl-Met-Leu-Phenylalanine), LPS (from *Escherichia coli* 0111:B4), HEPES solution, and L-glutamine were purchased from Sigma–Aldrich Chemie GmbH (Germany). Fetal bovine serum (FBS) and phosphate-buffered saline (PBS) were purchased from Gibco (USA). Ficoll Hypaque gradient (LSM 1077) was obtained from PAA Laboratories, GmbH (Austria). Human Quantikine ELISA Kits were purchased from R&D System (USA). Anti- pp38 (#9211), ppJNK (#9251), pJNK (#9252), pERK1/2 (#9102), NFκB(p65) (#8242S), and β-actin (#4967) antibodies were purchased from Cell Signaling Technology (USA). Anti- ppERK1/2 (#V8031) was purchased from Promega (USA) and anti-p38 (#SC-535) from Santa Cruz Biotechnology (USA). Propidium iodide was purchased from BD Biosciences (USA).

NMR spectra were recorded on a Bruker Advance III 600 MHz spectrometer (Bruker Biospin, Germany) in methanol-*d*_4_. Preparative HPLC was performed with a Shimadzu LC-20AP instrument (Japan) using a Zorbax SB-C18 column (150 × 21.2 mm, 5 μm) (Agilent, USA) at a flow rate of 20.0 mL/min. TLC was performed on Merck silica gel 60 F 254 (0.25 mm) plates with a dichloromethane/methanol/formic acid/water (80:25:1.5:4; v/v/v/v) solvent system. Chromatograms were visualized by spraying with sulfuric acid (5% in methanol) followed by heating at 105°C for 10 min. All solvents used for chromatography were of gradient grade. HPLC-DAD-MS^n^ analysis was performed on a UHPLC-3000 RS system (Dionex, Germany) with DAD detection and an AmaZon SL ion trap mass spectrometer with an ESI interface (Bruker Daltonik GmbH, Germany). Separation was performed on a Zorbax SB-C18 column (150 × 2.1 mm, 1.9 μm) (Agilent, USA). The mobile phase consisted of 0.1% HCOOH in water (A) and 0.1% HCOOH in MeCN (B) using the following gradients: 0–60 min, 5–40% B. The LC eluate was introduced into the ESI interface without splitting, and compounds were analyzed in negative ion mode with the following settings: nebulizer pressure of 40 psi; drying gas flow rate of 9 L/min; nitrogen gas temperature of 300°C; and a capillary voltage of 4.5 kV. The mass scan ranged from 100 to 2,200 m/z. UV spectra were recorded in the range of 200–400 nm.

The absorbance and luminescence were measured using a BioTek microplate reader (Highland Park, USA). Flow cytometry was performed using a BD FACSCalibur apparatus (BD Biosciences, USA).

### Plant material

**Figure 1:**
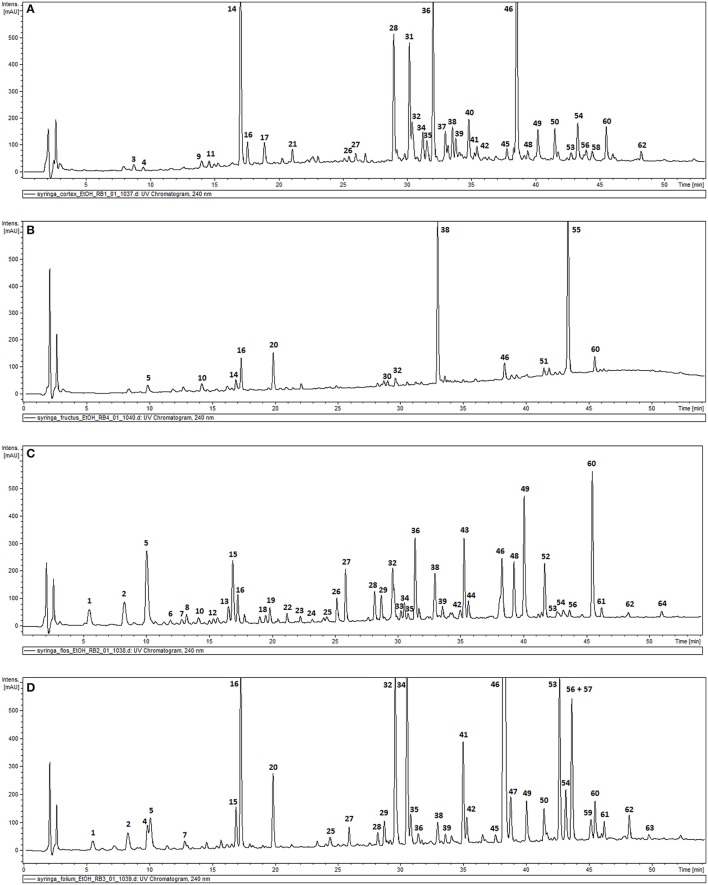
HPLC-DAD chromatograms of the ethanolic extracts of *S. vulgaris* bark **(A)**; fruit **(B)**; flower **(C)**; leaf **(D)** recorded at 240 nm.

Bark from the branches, leaves, flowers, and fruits of *S. vulgaris* L. were collected in May and August (fruits) 2016, from a native plant growing in Legionowo, Mazovian district, Poland (52° 23′ 17.647″ N; 20° 55′ 53.171″ E). The plant material was authenticated according to Flora Europaea (Tutin et al., 1972) by Anna K. Kiss. A voucher specimen (no. SV052015) has been deposited in the Plant Collection, Department of Pharmacognosy and Molecular Basis of Phytotherapy, Medical University of Warsaw, Poland (Figure 1S).

### Extract preparation and phytochemical characterization by HPLC-DAD-MS/MS

#### Infusions (aqueous extracts)

Air-dried plant materials (5 g) were poured with boiling water (250 mL), covered, and allowed to stand for 15 min. Extracts were then filtered and lyophilized, resulting in the following aqueous extract masses: bark- 0.52 g, fruit- 0.48 g, flower- 1.69 g, and leaf- 1.04 g.

#### Ethanolic extracts

The air-dried plant materials (2 g) were extracted with 60% ethanol (v/v, 1:20) in a water bath (70°C) for 1 h. Then, extracts were filtered, the ethanol was evaporated, and the water residues were lyophilized, resulting in the following ethanolic extract masses: bark- 0.25 g, fruit- 0.21 g, flower- 0.58 g and leaf- 0.51 g.

The extracts were characterized using an HPLC-DAD-MS/MS method. The presence of substances in extracts was confirmed by comparing the retention times and spectra (UV, MS, MS/MS) with standards and/or published data.

### Bioassay-guided isolation of active compounds from leaf extract

The air-dried plant material (100 g) was crushed and extracted three times with 75% methanol (1:10) at 70°C for 1 h each time (3 times). The methanol from combined extracts was evaporated under reduced pressure, and the aqueous residue was lyophilized to yield 40 g of extract. The crude extract was subjected to Diaion HP-20 (Supelco) column chromatography (45 × 5 cm) and eluted with an H_2_O-MeOH gradient (100:0 → 0:100, v/v) in 5 steps of 1.5 L each to obtain 35 fractions, which were pooled into 6 main fractions (F1-F6) based on their TLC and HPLC profiles. The activity of the fractions toward the inhibition of IL-8 and TNF-α was tested at a concentration of 50 μg/mL. Active fraction F6 (2.5 g) was subjected to further separation on a Sephadex LH-20 (Pharmacia) column (25 × 3 cm) with H_2_O-MeOH (50:50, v/v) to obtain 20 fractions of 50 mL each, which were pooled into 6 main fractions (F6_1–F6_6) based on their TLC and HPLC profiles. From fraction F6_2 (235 mg), compounds **I** (75 mg; Rt = 11.3–12.3 min.) and **II** (13 mg; Rt = 13.4–13.9); from fraction F6_3 (633 mg), compounds **III** (78 mg; Rt = 8.3–8.9 min) and **IV** (90 mg; Rt = 11.9–12.7 min), from fraction F6_4 (243 mg), compounds **V** (5 mg; Rt = 12.0–12.3 min), **VI** (23 mg; Rt = 15.4-16.0 min) and **VII** (7 mg; Rt = 19.0–19.5 min) were isolated using preparative HPLC with a gradient of 0.1% HCOOH in H_2_O-0.1% HCOOH in MeCN (80:20 → 40:60) over 60 min.

### Preparation of solutions of extracts, fractions, and compounds for bioassay

Tested extracts were dissolved in PBS or HBSS (1 mg/mL). Fractions and compounds were dissolved in DMSO (10 mg/mL or 10 mM stock solutions) and then diluted with RPMI 1640 medium. The extracts were tested in a concentration range of 25–100 μg/mL. Compounds were tested at concentrations of 10-50 μM. The concentration of DMSO used (<0.1%) did not influence the assays.

### Isolation of human neutrophils

Peripheral venous blood was taken from healthy human donors (18–35 years old) in the Warsaw Blood Donation Centre. Donors did not smoke or take any medications. They were clinically recognized to be healthy and a routine laboratory tests showed all values to be within the normal ranges. The study conformed to the principles of the Declaration of Helsinki.

Neutrophils were isolated by dextran sedimentation and centrifugation in a Ficoll Hypaque gradient (Böyum, 1968) and then re-suspended in HBSS buffer or RPMI 1640 medium.

### Cytotoxicity

Cytotoxicity was determined by a standard flow cytometric probe using propidium iodide (PI) staining. After 24 h of incubation with extracts/compounds, the neutrophils were harvested and centrifuged (1500 RPM; 10 min; 4°C), washed once with cold PBS and re-suspended in 400 μL of PBS. Five microliters of PI (50 μg/mL) solution was added to the cell suspensions. After 15 min of incubation at room temperature, cells were analyzed by flow cytometry, and 10,000 events were recorded per sample. Cells that displayed high permeability to PI were expressed as a percentage of PI(+) cells.

### ROS production

Neutrophils (3.5 × 10^5^) were incubated in HBSS buffer with 50 μL of the tested extracts and 50 μL of luminol (20 mM) in a 96-well plate. ROS production was initiated by the addition of f-MLP (0.1 μg/mL) to obtain a total volume of 200 μL/well. Chemiluminescence changes were measured over 40 min at intervals of 2 min in a microplate reader. The background chemiluminescence produced by non-stimulated cells was also determined. The percentage of inhibition was calculated by comparison to the control without the tested extracts at the maximum luminescence.

### IL-8, MMP-9, MCP-1, and TNF-α production

Neutrophils (2 × 10^6^) were cultured in 24-well plates in RPMI 1640 medium with 10% FBS, 10 mM HEPES, and 2 mM L-glutamine in the presence or absence of LPS (100 ng/mL) for 24 h at 37°C with 5% CO_2_ in the presence or absence of test extracts/fractions/compounds. After 24 h, the neutrophils were harvested and centrifuged (2000 RPM; 10 min; 4°C). The amount of released cytokines was measured by enzyme-linked immunosorbent assay (ELISA) following the manufacturer's instructions (BD Biosciences, USA). The effects on IL-8, MMP-9, MCP-1, and TNF-α production were calculated by comparing the percentages of the released agents to the stimulated control, which lacked the test extracts/fractions/compounds.

### Western blotting

Neutrophils (4 × 10^6^) were suspended in RPMI 1640 medium and incubated for 40 min at 37°C in the presence or absence of LPS (100 ng/mL) and in the presence or absence of the test compounds. They were then centrifuged at 1500 RPM for 10 min at 4°C. Cells were lysed in ice-cold buffer containing PBS, 5 mM EDTA, 1% Triton X-100, phosphatase and protease inhibitors, and the resulting lysates were centrifuged at 8,000 RPM for 15 min at 4°C. The proteins were separated by 12% SDS-PAGE. Proteins were transferred to nitrocellulose filters and immunoblotted with rabbit anti-p38MAPK, anti-ERK1/2, and anti-JNK at 1:1000 dilutions and a rabbit anti-actin polyclonal antibody at a 1:2000 dilution. Peroxidase-conjugated affinipure goat anti-rabbit antibody was used as a secondary antibody at a dilution of 1:10000. Finally, the blots were incubated with chemiluminescent substrate for the detection of HRP (Thermo-Scientific, USA) for 10–15 min. Western blots were quantified using the ImageJ 1.38 software after densitometric scanning of the bands.

### Statistical analysis

The results were expressed as the mean ± SEM of three independent experiments performed at least in duplicate. All analyses were performed using Statistica 9 software. The statistical significance of the differences between means was established by ANOVA with Dunnett's or Tukey's *post hoc* test. *P*-values below 0.05 were considered statistically significant.

## Results

### Phytochemical characterization and comparison of *S. vulgaris* from bark, fruit, flower and leaf infusions and ethanolic extracts

The phytochemical analysis of *S. vulgaris* bark, fruit, flower and leaf infusions and ethanolic extracts was performed using a HPLC-DAD-MS/MS method, which allowed for the identification or partial identification of 64 compounds (Table 1 and Figure 1) from the following groups: phenylethanoids (compounds **27**, **31**, **36**, **39**, and **42**), rare phenylethanoids esterified with an oleoside 11-methyl ester (compounds **43**, **49**, **50**, and **61**), flavonoids (compounds **32**, **33**, and **35**), other phenolic compounds (**1**–**3**, **5**–**14**, **17**, **18**, and **24**), lignans (compounds **21** and **37**), iridoids (compounds **34** and **41**), secoiridoids (compounds **4**, **15**, **16**, **20**, **26**, **28**-**30**, **38**, **40**, **44**–**46**, **48**, **51**–**57**, **60**, **62**, and **63**), together with eight unknown compounds. Compounds **1**, **2**, **5**, **7**, **14**–**16**, **27**–**29**, **34**, **36**, **38**, **41**, **43**–**46**, **48**–**50**, **52**–**54**, **61** were previously isolated as pure compounds from *S. vulgaris* leaves, flowers, and bark (Kurkin et al., 1989, 1990; Damtoft et al., 1995; Kikuchi et al., 2010; Dudek et al., 2017). However, this is a first comprehensive phytochemical analysis of a broad range of compounds from different parts of the plant. Significant differences were observed in the phytochemical profiles of different plant parts (Table 1 and Figure 1). In bark extracts, the major compounds were as follows: syringin (**14**; Rt = 16.9 min, *m*/*z* 417 [M-H+HCOOH]^−^), demethyloleuropein (**28**; Rt = 28.2 min, *m*/*z* 525 [M-H]^−^), forsythoside B (**31**; Rt = 30.0 min, *m*/*z* 755 [M-H]^−^), acteoside (**36**; Rt = 31.4 min, *m*/*z* 623 [M-H]^−^) and oleuropein (**46**; Rt = 38.3 min, *m*/*z* 539 [M-H]^−^). In fruit extracts, the major compounds were as follows: nuzhenide (**38**; Rt = 33.0 min, *m*/*z* 685 [M-H]^−^) and an oleonuezhenide isomer (**55**; Rt = 43.3 min, *m*/*z* 1071 [M-H]^−^). In flower extracts, the major compounds were as follows: caffeoylglucaric acid (**5**; Rt = 10.1 min, *m*/*z* 371 [M-H]^−^), quercetin rutinoside (**32**; Rt = 30.1 min, *m*/*z* 609 [M-H]^−^), acteoside (**36**; Rt = 31.4 min, *m*/*z* 623 [M-H]^−^), oleuropein (**46**; Rt = 38.3 min, *m*/*z* 539 [M-H]^−^), oleoacteoside (**49**; Rt = 40.1 min, *m*/*z* 1009 [M-H]^−^) and oleonuezhenide (**60**; Rt = 45.4 min, *m*/*z* 1071 [M-H]^−^). In leaf extracts, the major compounds were as follows: secologanoside (**16**; Rt = 17.3 min, *m*/*z* 389 [M-H]^−^), quercetin rutinoside (**32**; Rt = 30.1 min, *m*/*z* 609 [M-H]^−^), syringalactone B (**34**; Rt = 30.5 min, *m*/*z* 525 [M-H]^−^), oleuropein (**46**; Rt = 38.3 min, *m*/*z* 539 [M-H]^−^), 2″-epi-frameroside (**53**; Rt = 42.6 min, *m*/*z* 601 [M-H]^−^), and ligstroside (**54**; Rt = 43.1 min, *m*/*z* 523 [M-H]^−^). The phytochemical profiles of the infusions and ethanolic extracts were similar, and only quantitative differences were observed.

**Table 1:** Retention time, UV, and MS/MS data of the compounds present in extracts and infusions from *S. vulgaris*.

	**Compound**	**RT (min)**	**UV (nm)**	**[M-H]^−^*m*/z**	**Ion fragmentation**	**BE**	**BA**	**FrE**	**FrA**	**FLE**	**FLA**	**LE**	**LA**	**References**
1	Caffeoylglucaric acid (I)	5.5	326	371	353, **209**, 190	−	−	−	−	+	+	+	+	Dudek et al., 2017
2	Caffeoylglucaric acid (II)	8.3	325	371	353, **209**, 190	−	−	−	−	+	+	+	+	Dudek et al., 2017
3	Hydroxytyrosol hexoside	8.7	280	315	**135**	+	+	−	−	−	−	−	−	Bi et al., 2011
4	Oleoside	9.8	227	389	345, **227**, 209, 183, 165, 121	+	+	−	−	−	−	+	+	Li et al., 2017
5	Caffeoylglucaric acid (III)	10.1	325	371	353, **209**, 190	−	−	+	+	+	+	+	+	Dudek et al., 2017
6	*p*-coumaroylhexaric acid (I)	11.5	313	355	337, 209, **190**	−	−	−	−	+	+	−	−	Dudek et al., 2017
7	Caffeoylglucaric acid (IV)	12.9	325	371	353; 209, 190	−	−	−	−	+	+	+	+	Dudek et al., 2017
8	Caffeic acid hexoside (I)	13.3	325	341	251, 203, **179**, 161, 135	−	−	−	−	+	+	−	−	Fusani et al., 2016
9	Caffeic acid hexo–rhamnoside	14.1	335	487	**179**, 135	+	−	−	−	−	−	−	−	Sanz et al., 2012
10	*p*-coumaroylhexaric acid (II)	14.2	315	355	337, 209, **190**	−	−	+	+	+	+	−	−	Dudek et al., 2017
11	Caffeic acid hexoside (II)	14.6	332	341	**179**	+	−	−	−	−	−	−	−	Fusani et al., 2016
12	Feruloylhexaric acid (I)	15.5	326	385	367, 209, **190**	−	−	−	−	+	+	−	−	−
13	Feruloylhexaric acid (II)	16.5	326	385	367, 209, **190**	−	−	−	−	+	+	−	−	−
14	Syringin	16.9	265	417^*^	371, **209**	+	+	+	+	−	−	−	−	Tóth et al., 2016
15	Oleoside/secologanoside 11-methyl ester	17.0	225	403	371, **223**, 179	−	−	−	−	+	+	+	+	Li et al., 2017
16	Secologanoside	17.3	233	389	**345**, 209, 183, 165, 121	+	+	+	+	+	+	+	+	Li et al., 2017
17	Coniferaldehyde	18.9	343	177	−	+	+	−	−	−	−	−	−	Sanz et al., 2012
18	Ferulic acid hexoside	19.5	325	355	217, **193**	−	−	−	−	+	−	−	−	−
19	Unknown	19.8	233	447^*^	401	−	−	−	−	+	+	−	−	−
20	Oleoside/secologanoside 11-methyl ester	19.9	236	403	371, **223**, 179	−	−	+	+	−	−	+	+	Li et al., 2017
21	Olivil hexoside	21.1	278	583^*^	**537**, 345, **375**, 195, 179	+	−	−	−	−	−	−	−	Sanz et al., 2012
22	Unknown	21.3	212	377	**197**, 153	−	−	−	−	+	−	−	−	−
23	Unknown	22.2	312	687	315	−	−	−	−	+	+	−	−	−
24	Caffeic acid derivative	23.2	325	335	**179**, 135	−	−	−	−	+	+	−	−	−
25	Unknown	24.3	215	375	357, **191**, 129	−	−	−	+	+	+	+	+	−
26	Safghanoside C	25.1	222	671	**491**, 371, 299, 281, 191	+	−	−	−	+	+	−	−	Takenaka et al., 2002
27	Echinacoside	25.8	328	785	623, **477**, 461	+	−	−	−	+	+	+	−	Tóth et al., 2016
28	Demethyloleuropein	28.2	215	525	**481**, 389, **195**	+	+	−	−	+	+	+	+	Dudek et al., 2017
29	Oleoside dimethyl ester	28.6	320	463^*^	417, **255**, 185	−	−	−	−	+	+	+	−	−
30	10-hydroxyoleuropein	29.0	235	555	537, 403, 393, 322	−	−	+	+	−	−	−	+	Tóth et al., 2016
31	Forsythoside B	30.0	330	755	**593**, 461, 447, 315	+	+	−	−	−	−	−	−	Guo et al., 2007
32	Quercetin rutinoside	30.1	256, 355	609	**301**	+	+	+	+	+	+	+	+	Li et al., 2017
33	Kaempferol rutinoside	30.2	265, 341	593	**285**	−	−	−	−	+	+	−	−	Guo et al., 2007
34	Syringalactone B	30.5	228, 280	525	**363**, 249	+	+	−	+	+	+	+	+	Dudek et al., 2017
35	Quercetin hexoside	30.9	255, 353	463	**301**	+	+	−	−	+	−	+	+	−
36	Acteoside	31.4	330	623	**461**, 315, 161, 135	+	+	−	−	+	+	+	−	Tóth et al., 2016
37	Pinoresinol hexoside	32.9	278	519	**357**	+	−	−	−	−		−	−	Kicel et al., 2015
38	Nuzhenide	33.0	223	685	**523**, 453, 299	+	−	+	+	+	+	+	−	Dudek et al., 2017
39	Acteoside isomer	33.7	335	623	461, **315**	+	+	−	−	+	−	+	−	−
40	Nuzhenide isomer	34.8	275	685	**523**, 453, 299	+	+	−	−	−	−	−	−	−
41	Syringalactone A	35.1	223	509	**347**, 233, 207, 165	+	−	−	−	−	+	+	+	Dudek et al., 2017
42	Lipedoside A	35.2	324	607	**461**, 443, 315, 161, 135	+	+	−	−	+	−	+	+	He et al., 1994
43	Oleoechinacoside	35.4	334	1172	**1009**, 997, 785, 623	−	−	−	−	+	+	−	−	Dudek et al., 2017
44	Demethyloleoneonuezhenide	35.7	231	1074	**701**, 539	−	−	−	−	+	−	−	−	Dudek et al., 2017
45	Neonuezhenide	37.5	217	701	377, **307**, 275	+	−	−	−	−	−	+	−	Dudek et al., 2017
46	Oleuropein	38.3	280	539	**377**, 307, 275, 191	+	+	+	+	+	+	+	+	Dudek et al., 2017
47	Unknown	38.7	280	461	**419**, 363, 307	−	−	−	−	−	−	+	−	−
48	Demethyloleonuezhenide	39.1	215	1057	**685**, 523, **453**	+	+	−	−	+	+	−	−	Dudek et al., 2017
49	Oleoacteoside	40.1	330	1009	847, 745, 665, **623**, 461	+	+	−	−	+	+	+	−	Dudek et al., 2017
50	Isooleoakteoside	41.4	330	1009	847, **745**, 665, 623, 461	+	+	−	−	−	−	+	−	Dudek et al., 2017
51	Lucidumoside C	41.4	275	583	537, 403, 223	−	−	+	−	−	−	−	−	Li et al., 2017
52	Oleoneonuezhenide	41.6	222	1087	**701**, 539	−	−	−	−	+	+	−	−	Dudek et al., 2017
53	2″-epi-frameroside	42.6	235	601	403, **197**	+	+	−	+	+	+	+	+	Takenaka et al., 2002
54	Ligstroside	43.1	275	523	**361**, 291, 259	+	+	+	−	+	−	+	−	Dudek et al., 2017
55	Oleonuezhenide isomer	43.3	234	1071	**773**, 687, 523	−	−	+	+	−	−	−	−	−
56	Neooleuropein	43.5	215	661	429, 345	+	−	−	−	+	+	+	−	Kuwajima et al., 1992
57	Isoligustroside	43.6	275	523	**361**, 291	−	−	−	−	−	−	+	+	Kikuchi et al., 1987
58	Unknown	44.5	320	1167	−	+	−	−	−	−	−	−	−	−
59	Unknown	45.1	218	347	**233**, 209, 139	−	−	−	−	−	−	+	−	−
60	Oleonuezhenide	45.4	217	1071	1041, **771**, 685	+	+	+	+	+	+	+	−	Dudek et al., 2017
61	Syringaoleoacteoside	46.2	334	1395	1051, 1009, **565**	−	−	−	−	+	+	+	−	Dudek et al., 2017
62	Hydroxyframoside	48.1	275	645	**483**, 413, 345, 275	+	−	−	−	+	+	+	+	Iossifova et al., 1998;
63	Framoside	49.8	280	629	**491**, 329	−	−	−	−	−	−	+	−	Tanahashi et al., 1992
64	Unknown	50.9	217	271	177, **151**	−	−	−	−	+	−	−	−	−

* [M-H+HCOOH]^−^

***E**, ethanolic extract; **A**, infusion; **B**, bark; **Fr**, fruit; **FL**, flower; **L**, leaf. Bold, most abundant fragmentation ion*.

### Effect of bark, fruit, flower and leaf infusions and ethanolic extracts of *S. vulgaris* on the pro-inflammatory function of stimulated neutrophils

**Figure 2:**
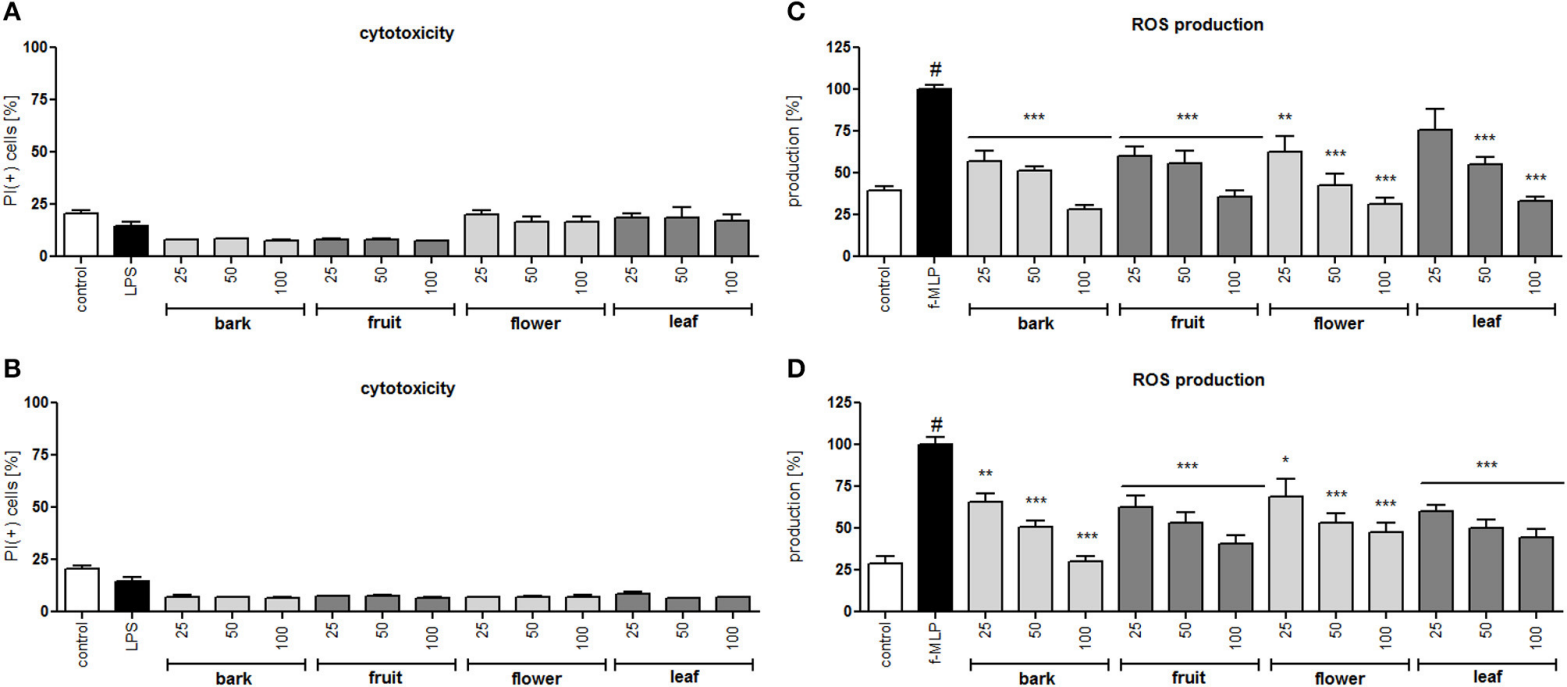
Effect of *S. vulgaris* ethanolic extracts **(A)** and infusions **(B)** at concentrations of 25–100 μg/mL on neutrophil membrane integrity {[%] of propidium iodide positive cells IP (+)}; effect of *S. vulgaris* ethanolic extracts **(C)** and infusions **(D)** at concentrations of 25–100 μg/mL on ROS production by stimulated neutrophils [%]. Data were expressed as the mean ± SEM; at least three independent experiments were conducted, and they were assayed in duplicate. Experiments were performed using cells from different donors. Statistical significance #*P* < 0.01 compared to the non-stimulated control; *P < 0.05, **P < 0.01, ***P < 0.001 decrease compared to the stimulated control.

**Figure 3:**
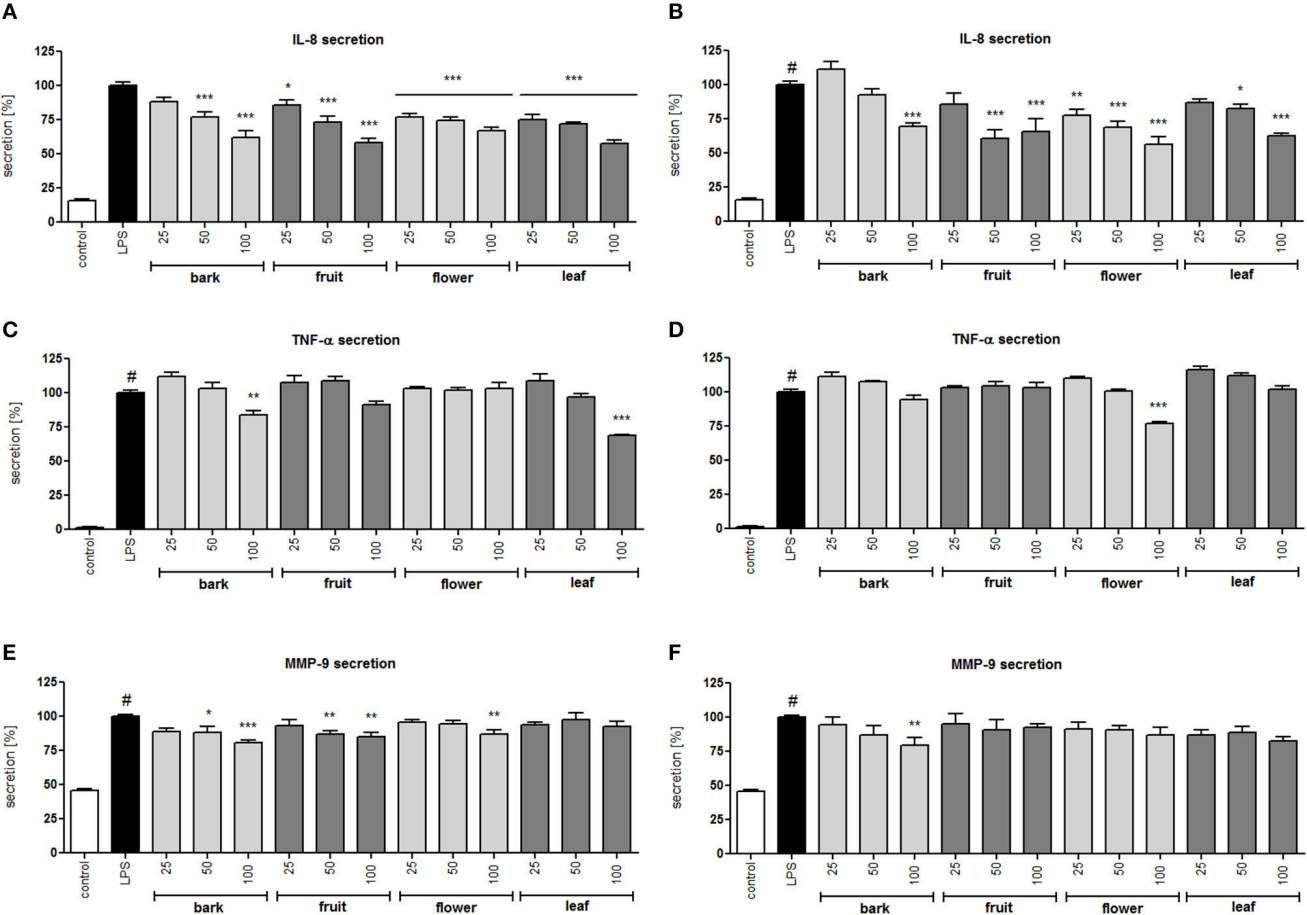
Effect of *S. vulgaris* ethanolic extracts **(A)** and infusions **(B)** at concentrations of 25–100 μg/mL on IL-8 production by stimulated neutrophils [%]; effect of *S. vulgaris* ethanolic extracts **(C)** and infusions **(D)** at concentrations of 25–100 μg/mL on TNF-α production by stimulated neutrophils [%]; effect of *S. vulgaris* ethanolic extracts **(E)** and infusions **(F)** at concentrations of 25–100 μg/mL on MMP-9 production by stimulated neutrophils [%]; Data were expressed as the mean ± SEM; at least three independent experiments were conducted, and they were assayed in duplicate. Experiments were performed using cells from different donors. Statistical significance #*P* < 0.01 compared to the non-stimulated control; *P < 0.05, **P < 0.01, ***P < 0.001 decrease compared to the stimulated control.

In all concentrations of the tested extracts, no significant reduction in membrane integrity was observed in comparison to the control using a propidium iodide assay (Figures 2A,B). Activation of neutrophils by f-MLP results in degranulation and a significant release of ROS compared to the untreated control (Figures 2C,D). Additionally, stimulation with LPS resulted in a significant release of proteinase MMP-9, chemokine IL-8, and cytokine TNF-α (Figure 3). Incubation of stimulated neutrophils with extracts at concentration ranges of 25–100 μg/mL resulted in a statistically significant and dose dependent reduction of ROS production by all extracts over all tested concentrations. There was no statistically significant difference between infusions and ethanolic extracts (Figures 2C,D).

All infusions and extracts were able to inhibit the release of IL-8 in a dose-dependent manner (Figures 3A,B). The effect was significant (*p* < 0.001) for all concentrations tested of the flower infusion and flower and leaf extracts. The effect on TNF-α production/release was less pronounced and was statically significant only at the highest concentration of 100 μg/mL for bark and leaf ethanolic extracts and the flower infusion (Figures 3C,D). MMP-9 release was inhibited the most by bark and fruit ethanolic extracts (Figures 3E,F). In general, ethanolic extracts were slightly more active than infusions, and flower and leaf preparations were more active than bark and fruit preparations. In a previous study, we isolated 29 compounds that were hydroxycinnamoyl and secoiridoid derivatives (Dudek et al., 2017) from the flower extract with moderate anti-inflammatory activity. In this study, we performed a bioassay-guided isolation of active compounds from the leaf extract.

### Bioassay-guided isolation of active compounds from leaf extract

**Figure 4:**
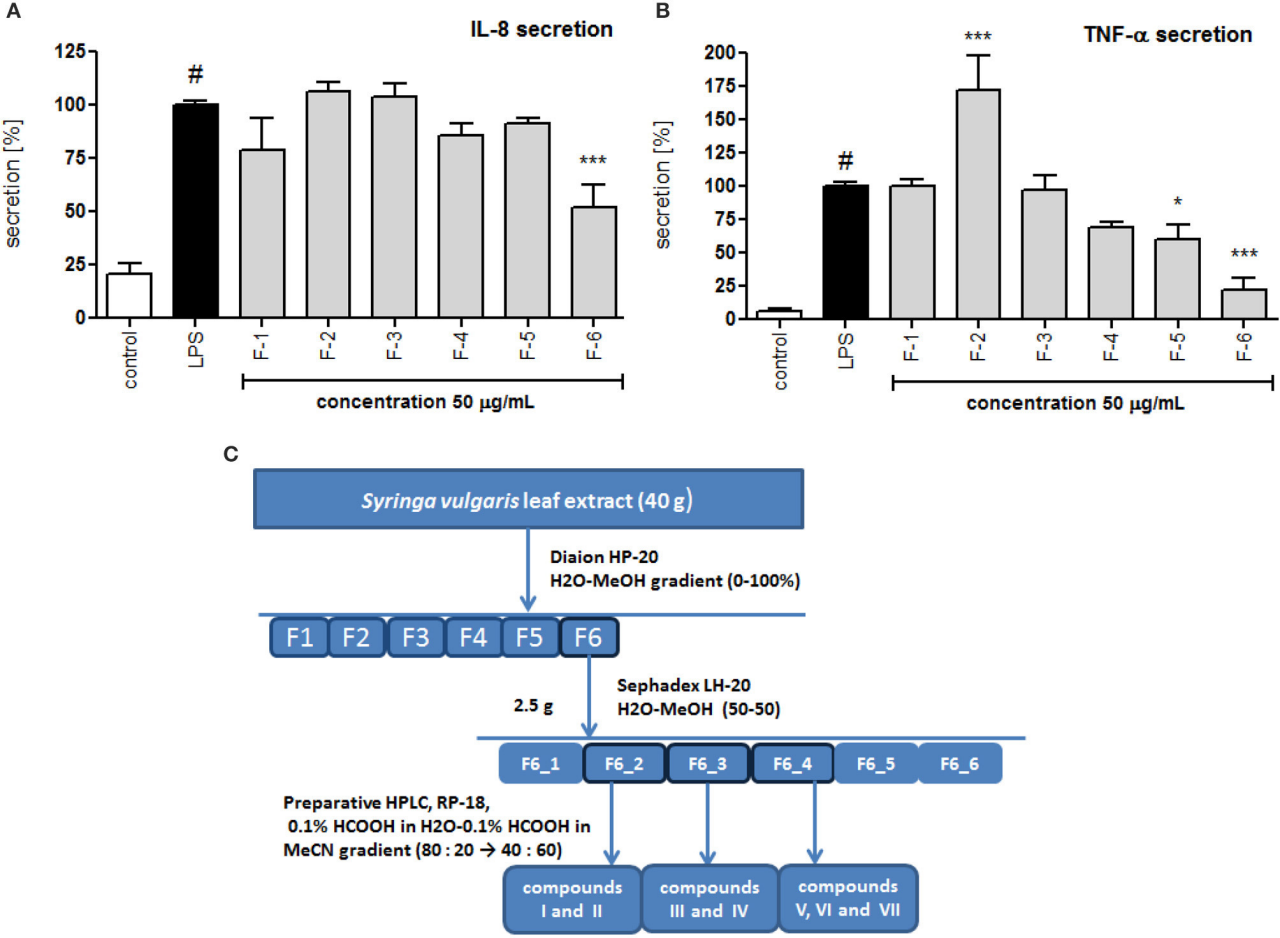
Effect of *S. vulgaris* leaf extract fractions on IL-8 production by stimulated neutrophils [%] **(A)** on TNF-α production by stimulated neutrophils [%] **(B)**; scheme of fractionation and compounds isolation from leaf extract **(C)**. Data were expressed as the mean ± SEM; at least three independent experiments were conducted, and they were assayed in duplicate. Experiments were performed using cells from different donors. Statistical significance #*P* < 0.01 compared to the non-stimulated control; *P < 0.05, **P < 0.01, ***P < 0.001 decrease compared to the stimulated control.

**Figure 5:**
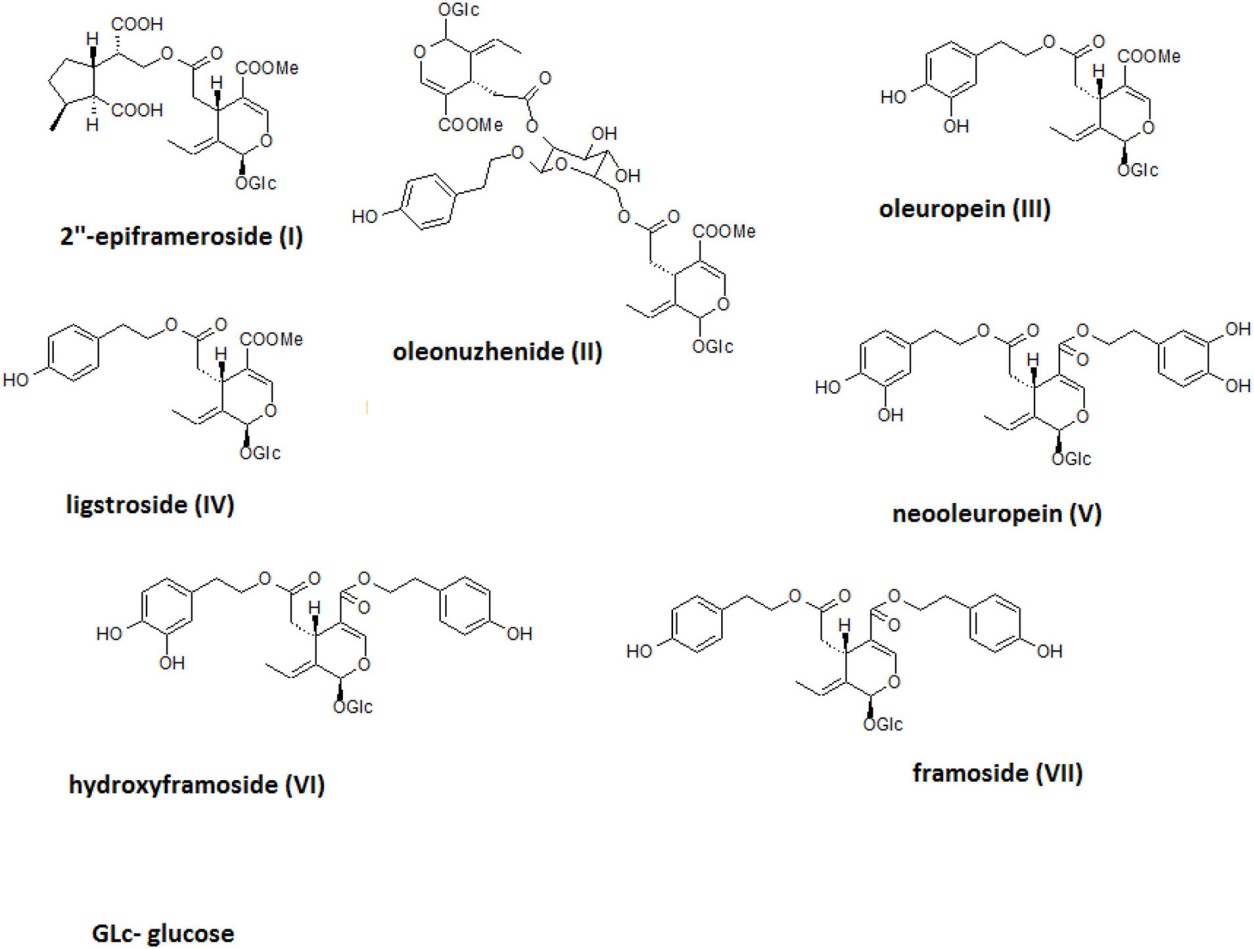
Structures of isolated compounds.

The crude alcoholic leaf extract was fractionated using a Diaion HP-20 and eluted with a 5-step H_2_O-MeOH gradient (100:0 → 0:100) to obtain six main fractions (F1-F6) based on their TLC and HPLC profiles. The activities of the fractions were evaluated based on IL-8 inhibition and TNF-α release. Only fraction F6 inhibited the IL-8 release at a concentration of 50 μg/mL in a statistically significant manner (*p* < 0.001). Interestingly, fraction F2 stimulated TNF-α production, while fractions F5 and F6 decreased the production of this cytokine (Figure 4). Fraction 5 was rich in oleuropein. Fraction 6, which contained several compounds, was subject to further fractionation on a Sephadex LH-20 with H_2_O-MeOH (50:50) and then preparative chromatography was used to isolate the following seven compounds: 2″-epiframeroside (**I**), oleonuezhenide (**II**), oleuropein (**III**), ligstroside (**IV**), neooleuropein (**V**), hydroxyframoside (**VI**), and framoside (**VII**) (Figure 4). The structures of these compounds (Figure 5) were confirmed based on their ^1^H and ^13^C NMR spectra, which were compared with reference data (Fukuyama et al., 1987; Damtoft et al., 1992; Kuwajima et al., 1992; Tanahashi et al., 1992; Iossifova et al., 1998; Konno et al., 1998; Takenaka et al., 2002). Analysis of the extracted ion intensity for single compounds in each extract confirmed that 2″-epiframeroside (**I**), oleuropein (**III**), ligstroside (**IV**), neooleuropein (**V**), hydroxyframoside (**VI**), and framoside (**VII**) were the major compounds present in leaf extract. Only oleonuezhenide (**II**) was present in a higher concentration in the flower extract (Figure 2S).

### Effect on cytokine/chemokine production, MAP kinases and NF-*k*B activation

**Figure 6:**
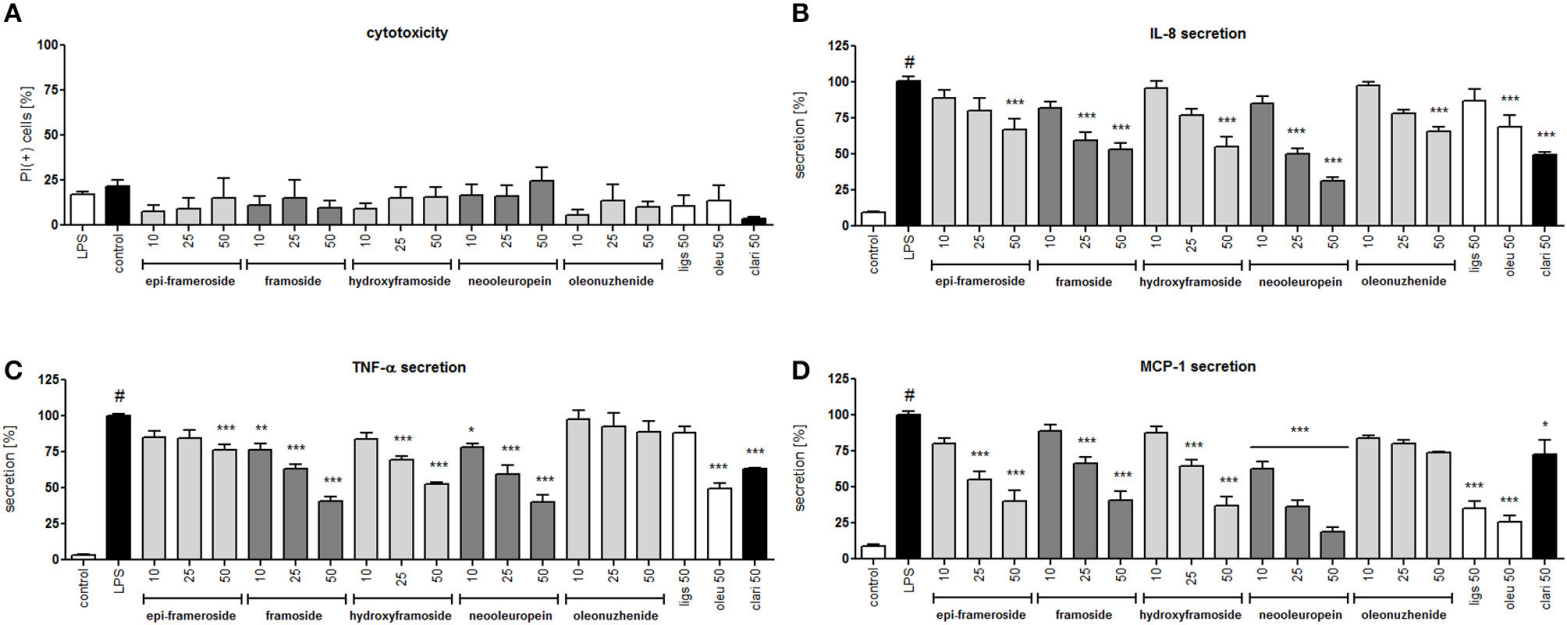
Effects of isolated compounds at concentrations of 10–50 μM, ligstroside (ligs), oleuropein (oleu) and a positive control clarithromycin (clari) at 50 μM: **(A)** membrane integrity {[%] of propidium iodide positive cells IP (+)}; **(B)** inhibition of IL-8 production from stimulated neutrophils [%]; **(C)** inhibition of TNF-α production from stimulated neutrophils [%]; **(D)** inhibition of MCP-1 production from stimulated neutrophils [%]; Data are expressed as the mean ± SEM; at least three independent experiments were performed, and they were assayed in duplicate. Experiments were performed using cells from different donors. Statistical significance #*P* < 0.01 compared to the non-stimulated control; *P < 0.05, **P < 0.01, ***P < 0.001 decrease compared to the stimulated control (LPS).

Lipopolysaccharide stimulation of human neutrophils resulted in the induction of the production/release of chemokines IL-8 (neutrophil chemotactic factor) and MCP-1 (monocyte chemoattractant protein 1) along with cytokine TNF-α in comparison to cells that were not stimulated. Isolated compounds **I**, **II**, **V**, **VI**, and **VII** were tested at concentrations of 10, 25, and 50 μM, and their activity was compared with oleuropein and ligstroside, the characteristic compounds of the Oleacea family that were also isolated from active fraction F6, and with the positive control clarithromycin, a macrolide antibiotic which has been shown to inhibit the pro-inflammatory function of neutrophils. At all concentrations of the tested compounds, no significant reduction in membrane integrity was observed in comparison to the control using a propidium iodide assay (Figure 6A). Neooleuropein was the most active compound toward inhibiting IL-8 secretion (Figure 6B); at concentrations of 25 and 50 μM, the release was reduced to 50.2% (*p* < 0.001) and 31.4% (*p* < 0.001) in comparison with LPS-stimulated cells (100% of release), respectively. The effect of neooleuropein at 50 μM was more significant than the effects of 2″-epiframeroside (*p* < 0.001), oleonuezhenide (*p* < 0.001), ligstroside (*p* < 0.001), and oleuropein (*p* < 0.001) at the same concentration. Framoside and neooleuropein were the most active in reduction of TNF-α release at all concentrations tested (Figure 6C). The effect of framoside at 50 μM was more significant than the effect of 2″-epiframeroside (*p* < 0.001), oleonuezhenide (*p* < 0.001), and ligstroside (*p* < 0.001) at the same concentration. Additionally, neooleuropein was statistically more active than the positive control clarithromycin (*p* < 0.05). All isolated compounds except oleonuezhenide significantly inhibited the release of MCP-1 from stimulated neutrophils (Figure 6D). However, only neooleuropein was active at all concentrations tested (Figure 6D). The effect of neooleuropein at 10 μM was more significant than the effects of framoside (*p* < 0.05) and hydroxyframoside (*p* < 0.05); at 25 μM, the effect was more significant than the effects of framoside (*p* < 0.01), hydroxyframoside (*p* < 0.01) and oleonuezhenide (*p* < 0.01), while at 50 μM, the effect was more significant than the effect of oleonuezhenide (*p* < 0.001) and the positive control clarithromycin (*p* < 0.01) at the same concentration. In general, neooleuropein was as effective or more effective in comparison with oleuropein and clarithromycin in reducing the release of pro-inflammatory agents.

**Figure 7:**
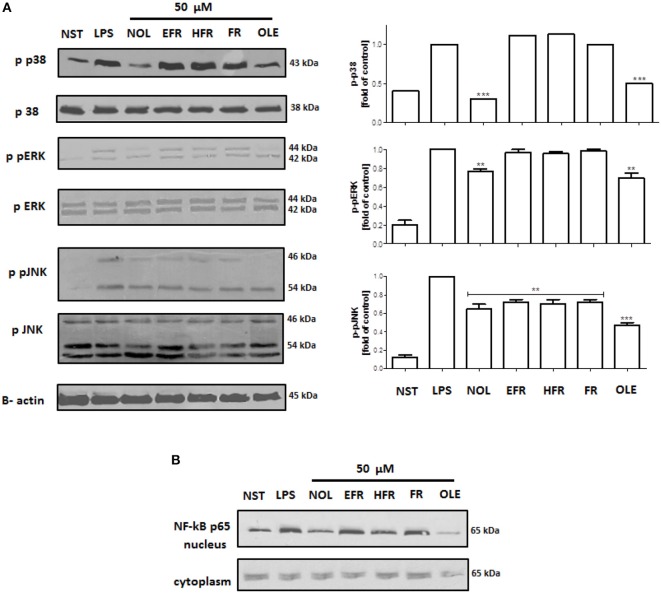
Effects of compounds at 50 μM on the phosphorylation of p38 MAPK, p42/44 ERK and JNK in LPS-activated human neutrophils **(A)**; and NF-*k*B p65 translocation to the nucleus **(B)**. Phosphorylation of p38, p42/44 ERK and JNK, and NF-*k*B p65 translocation were analyzed by an immunoblot assay using antibodies against the phosphorylated form and based on total protein. Representative images are shown (left **A,B**). Western blots for p38 MAPK, p42/44 ERK and JNK were quantified using the ImageJ 1.38 software after densitometric scanning of the bands of at least two independent experiments. Statistical significance **P < 0.01, ***P < 0.001 decrease compared to the stimulated control (right **A**). NST- not stimulated cells, LPS- stimulated cells, neooleuropein (NOL), 2″-epiframeroside (EFR), hydroxyframoside (HFR), framoside (FR), oleuropein (OLE).

The molecular mechanism of the observed effect was related to MAP kinases and NF-*k*B activation. Lipopolysaccharide stimulation of human neutrophils resulted in the rapid phosphorylation of proteins, including p38 MAPK, p42/44 extracellular signal-regulated kinase (ERK), and c-Jun NH_2_-terminal kinase (JNK), as well as the translocation of NF-*k*B-p65 from the cytoplasm to the nucleus. Our data show that the LPS-induced phosphorylation of p38 MAPK and ERK1/2 was decreased by neooleuropein and oleuropein, while JNK phosphorylation was decreased by all tested compounds at 50 μM (Figure 7A). The inhibition of the translocation of NF-*k*B-p65 from the cytoplasm to the nucleus was observed most significantly for oleuropein; however, the activity of neooleuropein was evident but more modest (Figure 7B).

## Discussion

*S. vulgaris* preparations have been traditionally used in Europe to treat several ailments connected with inflammation and, together with Asian *Syringa* sp., appear to be an interesting source of diverse bioactive structures (Su et al., 2015). The aim of the present study was to investigate the effects of phytochemically characterized (HPLC-DAD-MS^n^) extracts that have been used in traditional medicine (infusions and ethanolic extracts) from different parts of the common lilac (bark, fruits, leaves, and flowers) on the pro-inflammatory functions of neutrophils and to isolate the most active compounds using bioassay-guided fractionation. Finally, we focused on the activity and molecular mechanisms of action of the isolated compounds able to regulate neutrophil inflammation.

For the first time, we provided a comprehensive phytochemical analysis of common lilac and a comparison of different parts such as bark, fruit, flower, and leaf infusions/extracts (Table 1, Figure 1). We were able to identify or partially identify 64 compounds, and we also demonstrated significant differences in the phytochemical profiles of different parts of the plant (Figure 1). In general, the major compounds in *S. vulgaris* are as follows: caffeoylglucaric acid (**5**), syringin (**14**), secologanoside (**15**), demethyloleuropein (**28**), forsythoside B (**31**), quercetin rutinoside (**32**), syringalactone B (**34**), acteoside (**36**), nuzhenide (**38**), oleuropein (**46**), oleoacteoside (**49**), 2″-epi-frameroside (**53**), ligstroside (**54**), oleonuezhenide isomer (**55**), and oleonuezhenide (**60**). The presence of phenyl ethanoids **31**, **36**, **49**, and secoiridoids **28**, **38**, **46**, **53**, **54**, **55**, **60** as major compounds seems to be specific to this species, while lignans are more characteristic of *S*. *pinnatifolia* (Su et al., 2016), and secoiridoids named safghanosides are more characteristic of *S*. *afghanica* (Takenaka et al., 2002). *S. reticulata* contains similar phenyl ethanoids and secoiridoids as *S. vulgaris*; however, the presence of the iridoid syringapicroside and its derivatives in *S. reticulata* may distinguish both species phytochemically (Kikuchi et al., 1989; Machida et al., 2002).

In the neutrophil model of the pro-inflammatory state, all extracts and infusions were able to significantly reduce ROS production (Figures 2C,D). This is probably related to the presence of compounds that contain caffeic acid, *p*-coumaric acid, hydroxytyrosol or tyrosol phenolic moieties, which display antioxidant properties (Bi et al., 2011; Dudek et al., 2017). All extracts and infusions were especially active in decreasing IL-8 production (Figures 3A,B) and, to a lesser extent, MMP-9 and TNFα release (Figures 3C–F). However, the leaf extract fractionation revealed that fraction F2 increased TNFα production, while fractions F5 and F6 significantly decreased the production of this cytokine (Figure 4). A similar phenomenon was observed for *Echinacea purpurea* extracts (Todd et al., 2015). Further bioassay-guided fractionation of fraction F6 led to the isolation of the following secoiridoids: 2″-epiframeroside, oleonuezhenide, oleuropein, ligstroside, neooleuropein, hydroxyframoside, and framoside (Figure 5). We compared the anti-inflammatory activity of 2″-epiframeroside, oleonuezhenide, neooleuropein, hydroxyframoside, and framoside with oleuropein and ligstroside, two well-known compounds that are widespread in the Oleaceae family (Jensen et al., 2002). Apart from oleonuezhenide, a dimeric secoiridoid, all compounds exhibited significant inhibition of pro-inflammatory cytokine and chemokine release/production. 2″-epiframeroside primarily inhibited MCP-1 release, while neooleuropein appeared as the most active compound (Figure 6). Neooleuropein inhibited the secretion of IL-8 more significantly than oleuropein (Figure 6B) and secretion of TNF-α and MCP-1 more significantly than the positive control clarithromycin (Figures 6C,D).

The mechanism of LPS stimulation of human neutrophils is connected with a functional response through the activation of mitogen-activated protein kinases (MAPKs): p38 kinase, p42/44 extracellular signal-regulated kinase (ERK) and c-Jun NH_2_-terminal kinases (JNKs) (Nick et al., 1999; Arndt et al., 2004; Simard et al., 2015). Neooleuropein and oleuropein significantly inhibited the phosphorylation of the MAP kinases ERK1/2, p38 and JNK after LPS stimulation (Figure 7A), as well as the translocation of NF-*k*B p65 to the nucleus (Figure 7B). Neooleuropein was more active toward the inhibition of p38 phosphorylation, while oleuropein more significantly inhibited NF-*k*B p65 translocation, and all tested compounds inhibited JNK phosphorylation (Figures 7A,B). The activation of p38 MAPK in neutrophils is connected with the synthesis of TNF-α and IL-8 (Nick et al., 1999). ERK activation also led to the elevated expression of pro-inflammatory cytokines in human neutrophils (Simard et al., 2015). In our study, neooleuropein was the most active compound in inhibiting p38 phosphorylation, which correlated with a significant inhibition of IL-8 production. However, oleuropein was more active in NF-*k*B inhibition, which has been shown to affect cytokine gene expression in human neutrophils, although it has a less marked effect on IL-8 gene expression (Cloutier et al., 2007). The inhibition of MCP-1 production by all tested compounds was correlated with the inhibition of JNK phosphorylation, which has been strictly correlated with MCP-1 expression but not IL-8 or TNF-α expression (Arndt et al., 2004). Interestingly, framoside was able to significantly decrease the TNF-α and IL-8 secretion (Figures 6B,C) without affecting MAPKs nor NF-*k*B activation (Figure 7). It appears that framoside, the most lipophilic compounds, displays a different mode of action. As in human neutrophils other pathways such as PI3K are also involved in IL-8 production and acts downstream of p38 MAPK (Fortin et al., 2011).

The observed significant inhibition of TNF-α and chemokine (IL-8 and MCP-1) production is of special interest for treating inflammatory diseases such as rheumatic arthritis, respiratory diseases, and arteriosclerosis. The pro-inflammatory effect of TNF-α mainly results from its capacity to stimulate the expression of adhesive molecules in endothelial cells and promote neutrophil attachment to the vascular endothelium in addition to their degranulation and pro-oxidative activity (Witko-Sarsat et al., 2000). MCP-1 (CCL2) is able to stimulate chemotaxis of monocytes and cellular events associated with chemotaxis and integrin expression. At high concentrations, MCP-1 elicits a respiratory burst leading to the generation of ROS (Palomino and Marti, 2015). IL-8 (CXCL8), one of the most important cytokines produced by neutrophils, mediates chemotaxis, releases granule enzymes, and promotes integrin expression and adhesion to endothelial cells (Gabrilovich, 2013). Although the anti-inflammatory and antioxidant activity of oleuropein and ligstroside was intensively studied, this is the first report concerning the biological activity of 2″-epiframeroside, neooleuropein, hydroxyframoside, and framoside. In particular, neooleuropein appears to an interesting compound for further *in vitro* and *in vivo* study.

## Conclusions

The present study demonstrated that the common lilac, which has been traditionally used in Europe as a medicinal plant, is a valuable source of active compounds, especially neooleuropein, for further research regarding their use in treating inflammatory diseases that result from the excessive activation of neutrophils. The observed decreases in the production of cytokines, such as TNF-α, IL-8, MCP-1, depend on the inhibition of the phosphorylation of MAP kinases.

## Author contributions

MW, BM, MD, JW, and AK: performed the experiments; MW, BM, MD, and AK: carried out data analysis; AK: planned the experiments; AK: wrote the manuscript and supervised all work. All authors revised and approved the final version of the manuscript.

### Conflict of interest statement

The authors declare that the research was conducted in the absence of any commercial or financial relationships that could be construed as a potential conflict of interest.
