# Supplementary material for: Effects of Phytochemically Characterized Extracts From Syringa vulgaris and Isolated Secoiridoids on Mediators of Inflammation in a Human Neutrophil Model
Source: Front Pharmacol. 2018 Apr 11;9:349. doi: 10.3389/fphar.2018.00349 (PMC5904404; doi:10.3389/fphar.2018.00349)

**Supplementary materials**

**Effects of Phytochemically Characterized Extracts from *Syringa vulgaris* and Isolated Secoiridoids on Mediators of Inflammation in a Human Neutrophil Model**

Marta Woźniak^a^, Barbara Michalak^a^, Joanna Wyszomirska^a^, Marta K. Dudek^b^, Anna K. Kiss^a^*

^a^ Department of Pharmacognosy and Molecular Basis of Phytotherapy, Medical University of Warsaw, Banacha 1, 02-097, Warsaw, Poland

^b^ Centre of Molecular and Macromolecular Studies of Polish Academy of Sciences, Sienkiewicza 112, 90-363, Lodz, Poland

* Corresponding author. Medical University of Warsaw, Banacha 1, 02-097 Warsaw, Poland. Tel./fax: +48 22 572 09 85

E-mail address: akiss@wum.edu.pl

**Figure 1S.** *Syringa vulgaris L.*

**
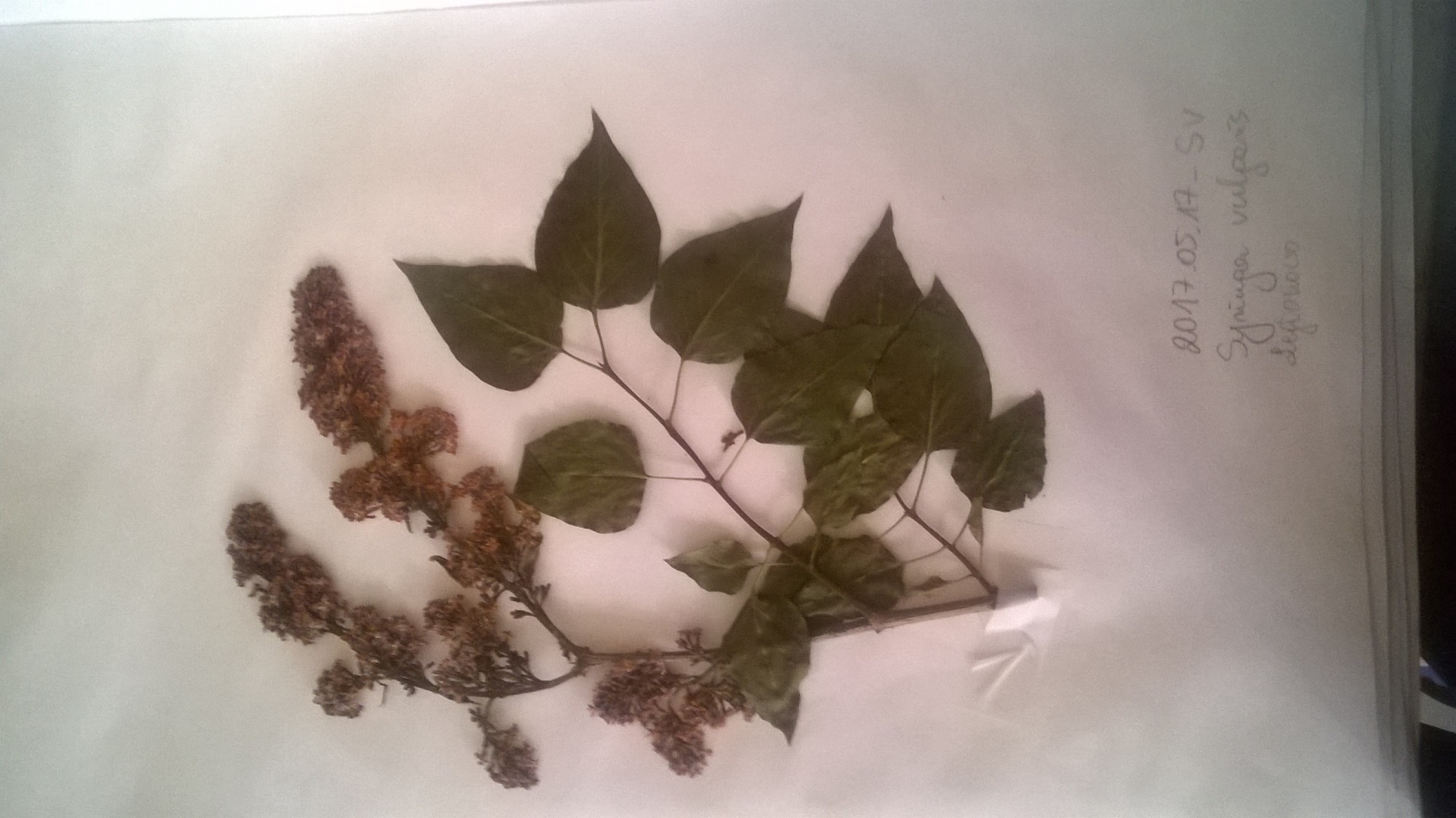
**

**Figure 2S.**

Extracted ion intensity for single compounds in each extract: **A**) 2’’-epiframeroside (**1**); **B**) oleonuezhenide (**2**); **C**) oleuropein (**3)**; **D**) ligstroside (**4**); **E**) neooleuropein (**5)**; **F**) hydroxyframoside (**6**); **G**) framoside (**7**)


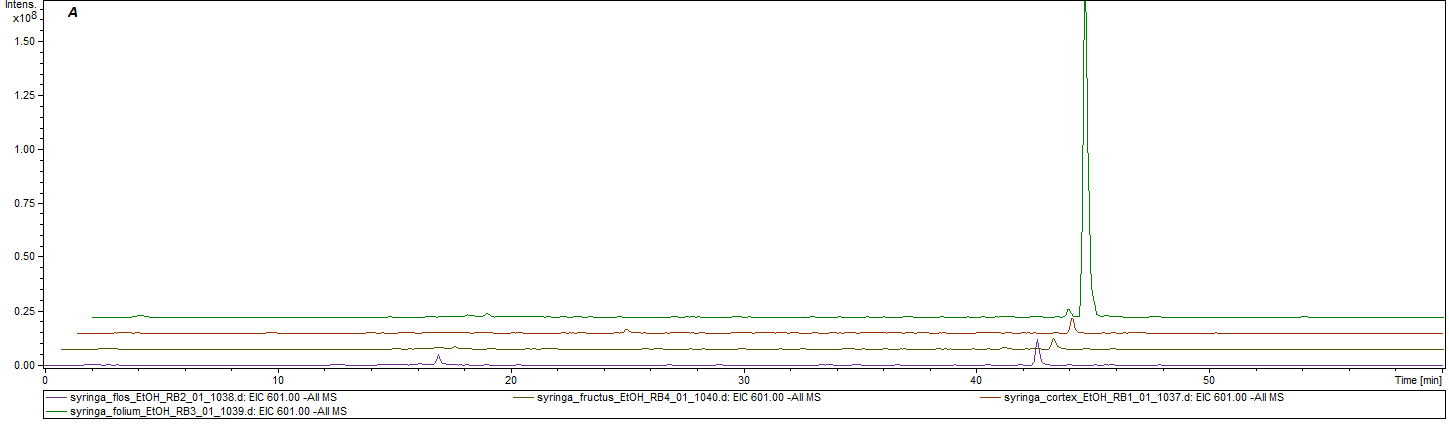


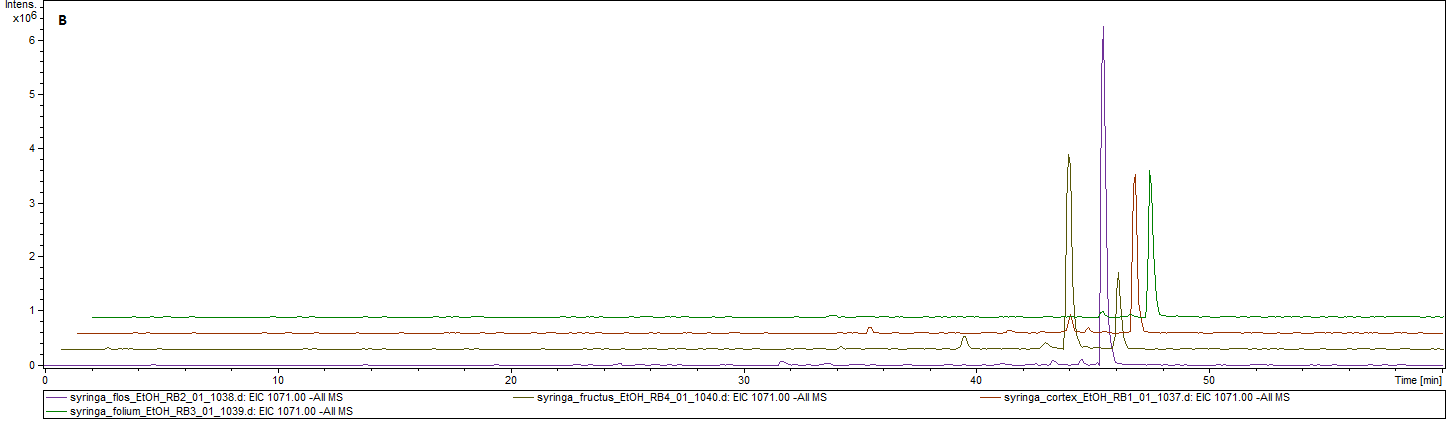

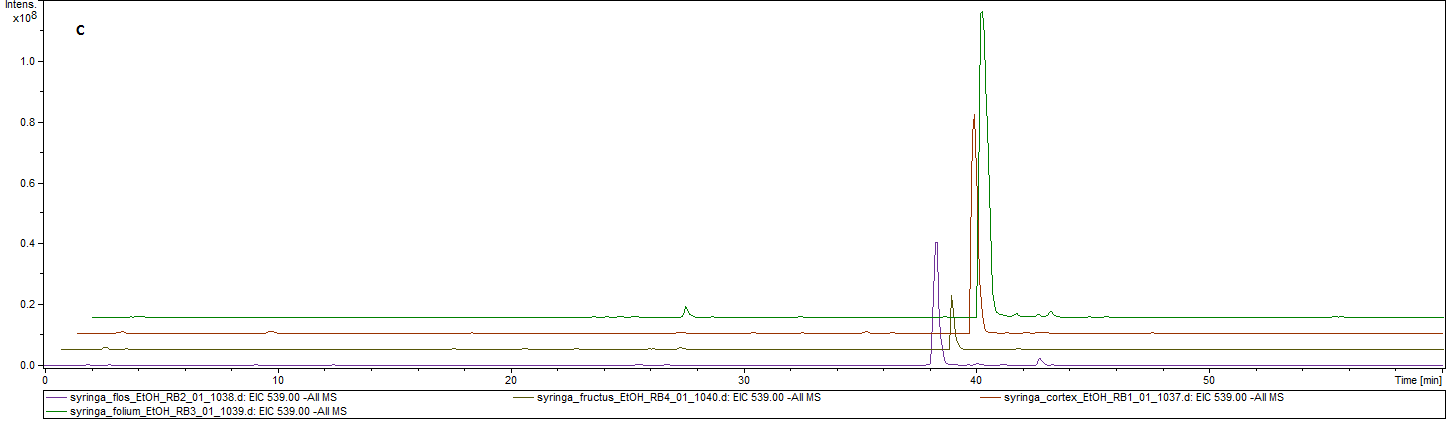

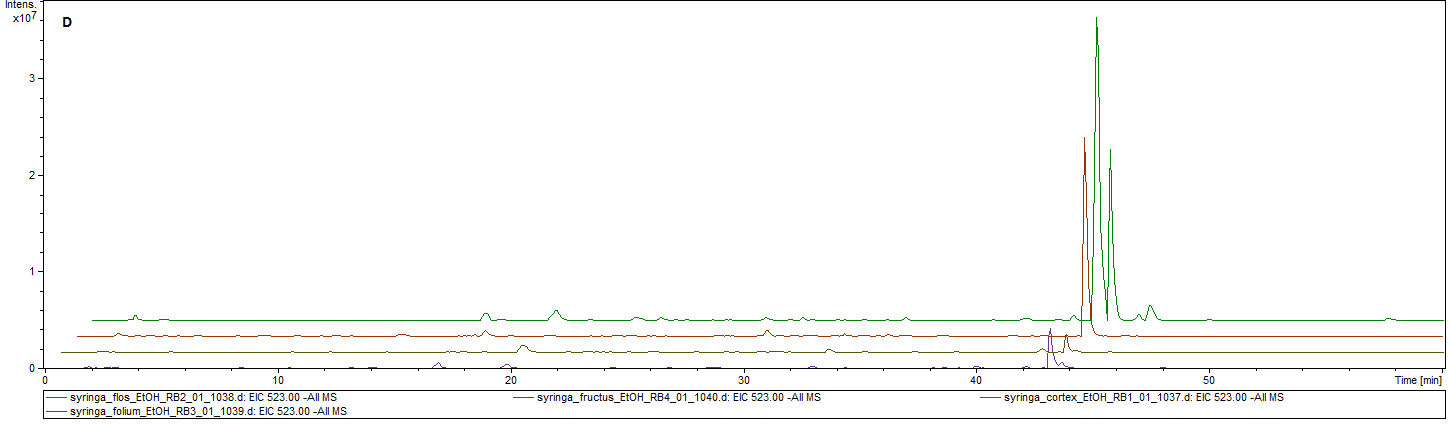

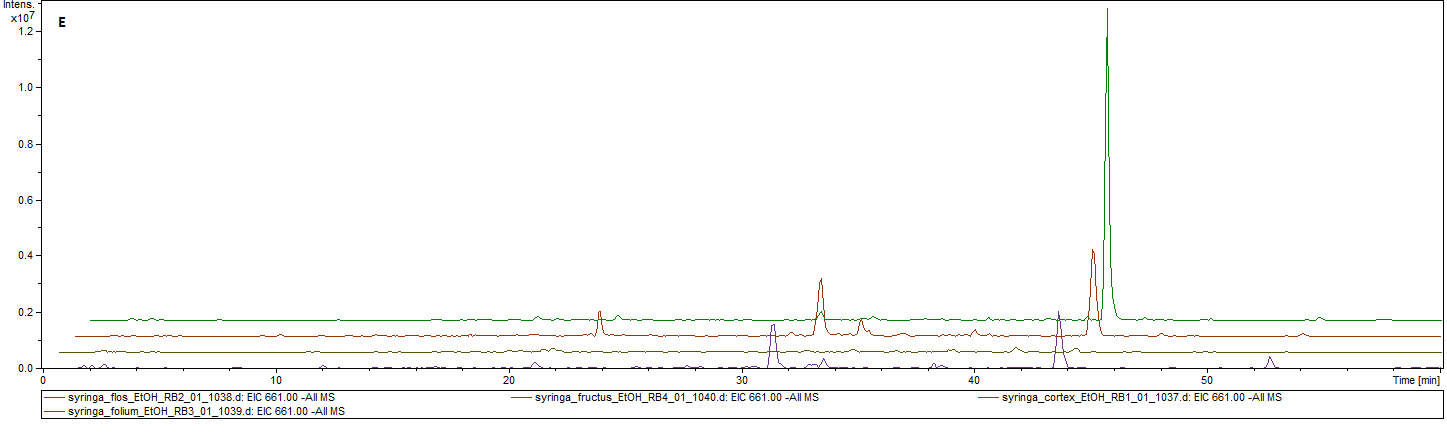

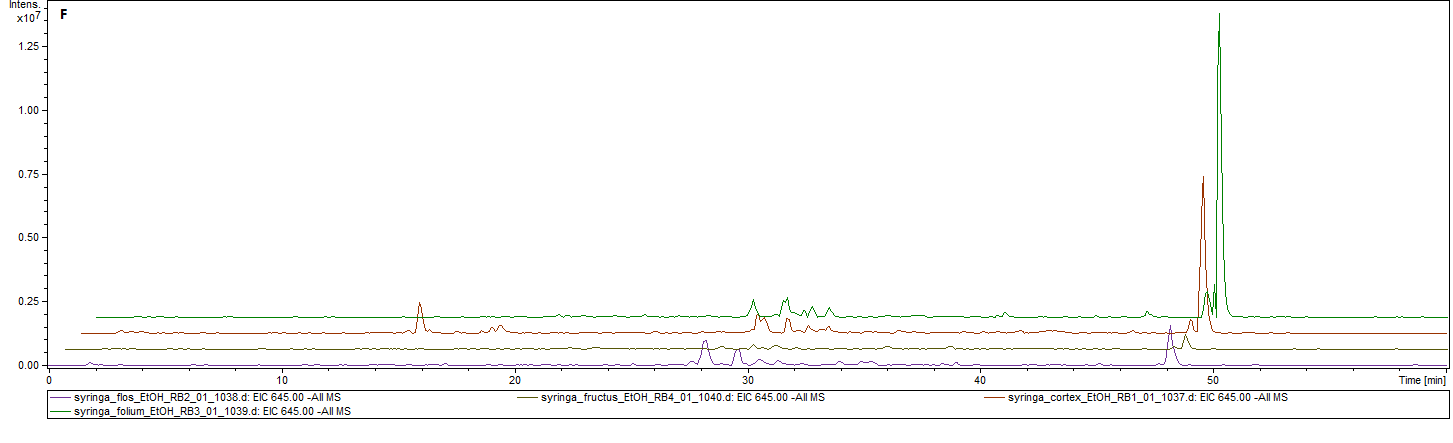

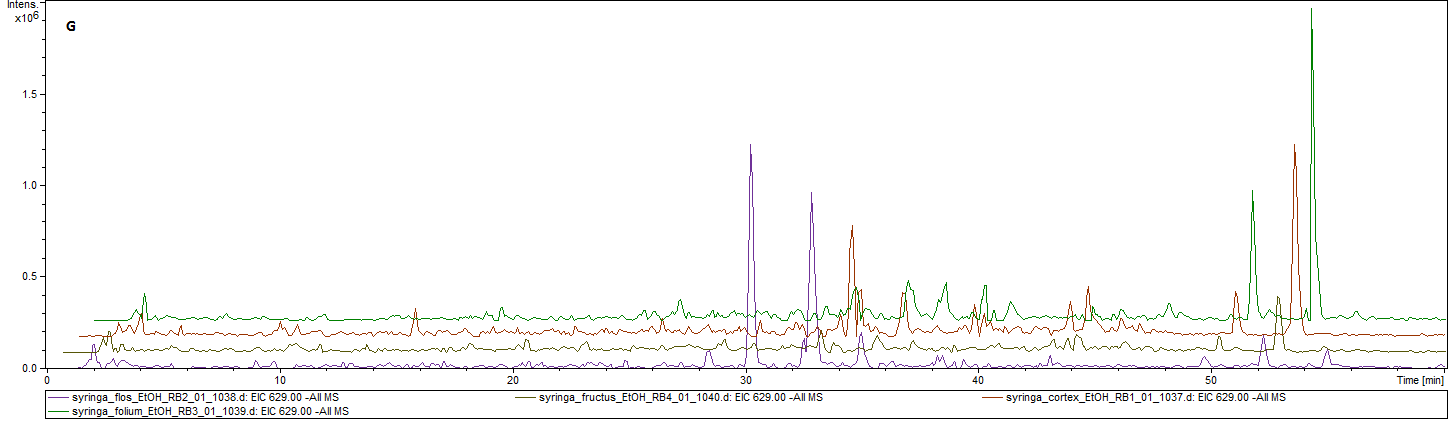

Supplement: Supplementary file 1 [file DataSheet1.docx]
